# Diabetic Muscle Infarction: A Rare Complication of Long-Standing and Poorly Controlled Diabetes Mellitus

**DOI:** 10.1155/2011/407921

**Published:** 2011-10-09

**Authors:** Shridhar N. Iyer, Almond J. Drake, R. Lee West, Robert J. Tanenberg

**Affiliations:** ^1^Division of General Internal Medicine, Department of Internal Medicine, The Brody School of Medicine, East Carolina University, Greenville, NC 27834, USA; ^2^Division of Endocrinology, Department of Internal Medicine, The Brody School of Medicine, East Carolina University, Greenville, NC 27834, USA; ^3^Department of Pathology, The Brody School of Medicine, East Carolina University, Greenville, NC 27834, USA

## Abstract

*Objective*. To report a case of diabetic muscle infarction (DMI), a rare complication of long-standing poorly controlled diabetes mellitus. *Methods*. We describe a case of a 45-year-old male with an approximately 8-year history of poorly controlled type 2 diabetes mellitus with multiple microvascular complications who presented with the sudden onset of left thigh pain and swelling. He had a swollen left thigh and a CK of 1670 U/L. He was initially treated with intravenous antibiotics for a presumptive diagnosis of pyomyositis or necrotizing fasciitis with no improvement. A diagnosis of diabetic muscle infarction was considered. *Results*. An MRI of the thigh demonstrated diffuse edema in the anterior compartment. A muscle biopsy demonstrated coagulation necrosis in skeletal muscle and inflammation and infarction in the walls of small blood vessels. These studies confirmed the final diagnosis of DMI. He was treated with supportive care and gradually improved. *Conclusion*. DMI is a rare complication of diabetes that is often mistaken for infections such as pyomyositis and necrotizing fasciitis or thrombophlebitis. Treatment is supportive. Although the short-term prognosis is good in these patients, the long-term prognosis is poor.

## 1. Introduction

Spontaneous diabetic muscle infarction (DMI) is a rare complication associated with uncontrolled diabetes mellitus (DM). These patients typically present with a palpable mass in an extremity, with localized pain and swelling. The patients usually have coexisting microvascular complications such as neuropathy, nephropathy, and retinopathy. Failure to recognize the condition often leads to unnecessary invasive procedures to establish a diagnosis. We describe a case of DMI and discuss its clinical features, pathogenesis, diagnosis, management, and prognosis.

## 2. Case Report

A 45-year-old African-American male with uncontrolled type 2 diabetes mellitus was admitted to a community hospital with sudden onset of pain in his left thigh associated with swelling. His past medical history included stage IV chronic kidney disease, background retinopathy, peripheral vascular disease requiring below the knee amputation of his left lower extremity, severe peripheral neuropathy, uncontrolled hypertension, and a prior history of tobacco use.

**Figure 1:**
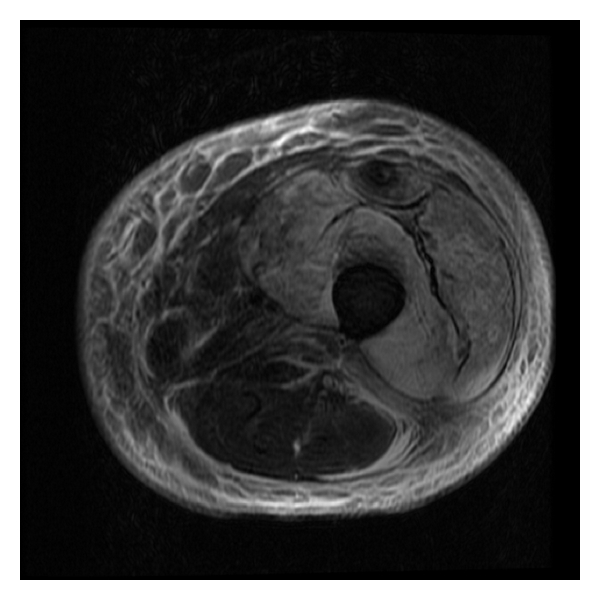
T_2_-weighted magnetic resonance image: transverse view of the left thigh: demonstrating diffuse edema in the anterior compartment and subcutaneous edema in the anterior and lateral aspects of the thigh.

He denied any trauma or administering insulin injections into his left thigh. He was noted to have an elevated creatine kinase of 1670 U/L (reference range, 32–294) and found to be severely anemic requiring transfusion with 4 units of packed red blood cells (PRBC). CKMB and troponin levels were 0.31 ng/mL (reference range, 0–5 ng/mL) and 0.02 ng/mL (reference range, 0–012 ng/mL), respectively. EKG demonstrated normal sinus rhythm with no ST-T changes. In addition, he was treated with intravenous antibiotics at presentation due to concerns for necrotizing fasciitis with no improvement in his condition. An MRI of the left thigh without contrast was obtained that demonstrated diffuse edema in the anterior compartment and subcutaneous edema in the anterior and lateral aspects of the thigh (Figure 1). Due to persistent symptoms, a biopsy of the left thigh muscle was done. In addition, he was also evaluated by a rheumatologist for a possible underlying autoimmune process, which was subsequently ruled out. Approximately 14 days after his initial presentation, he was transferred to our tertiary care medical center. On physical examination after transfer, the left thigh was swollen and firm, extending anteromedially to the lateral aspect of the thigh at the surgical biopsy site superiorly and to the left inguinal ligament. He had an incision measuring approximately 5 cm on the lateral aspect of his left thigh that was packed with gauze. The incision site was tender to palpation. Monofilament examination of his right lower extremity was compatible with diabetic polyneuropathy with loss of protective sensation.

**Figure 2:**
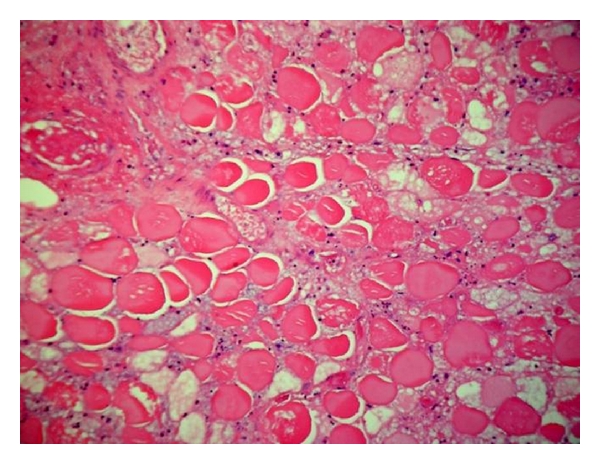
Histologic appearance of muscle tissue demonstrating coagulation necrosis in skeletal muscle (Hematoxylin-eosin stain: original magnification 200X).

**Figure 3:**
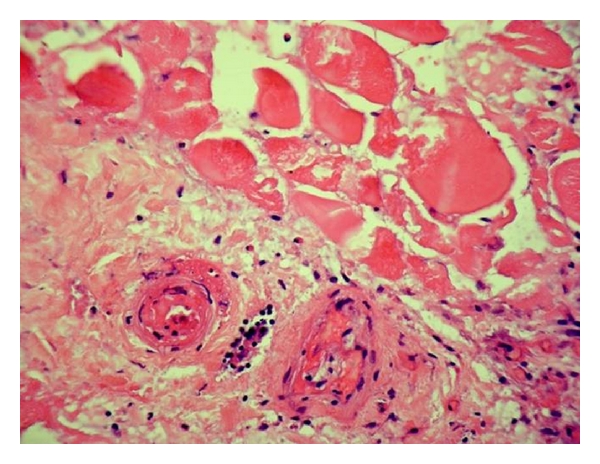
Histologic appearance of muscle tissue demonstrating small blood vessels in area of infarction with inflammation in the walls and thrombotic material in the lumen (Hematoxylin-eosin stain: original magnification 400X).

Laboratory studies at our hospital demonstrated a white blood cell count of 13 k/uL (reference range, 4.5–11.0), hemoglobin 10.6 g/dL (reference range, 13.0–18.0), hematocrit 31% (reference range, 40–52), serum creatinine of 4 mg/dL(reference range, 0.60–1.20), ESR 120 mm/h (reference range, 0–12), and a CRP was 213 mg/L (reference range, <2.6). An A_1_C of 7.1% (reference range, 4.3–5.7) was obtained after he was transfused 4 units of PRBC. An antinuclear antibody screen was within normal limits. A left lower extremity venous doppler revealed no evidence of deep venous thrombosis. Biopsy findings of the skin and subcutaneous tissue (reviewed independently by a surgical pathologist at our institution) demonstrated acute to subacute inflammation in the deep subcutaneous tissue and fascia. Biopsy of the left thigh muscle demonstrated coagulation necrosis with acute to subacute inflammation and areas of early fibrosis in skeletal muscle (Figure 2). Thrombotic material was seen in a few small blood vessels and the walls of some of the blood vessels were acutely inflamed (Figure 3).

The swelling and hardness gradually improved over the next week during which the patient rested his leg and was managed symptomatically for his pain. A week after admission at our institution he requested to be discharged. At the time of discharge his symptoms had improved but had not resolved completely. Since then he was lost to followup.

## 3. Discussion

DMI is a rare complication usually reported in association with long-standing poorly controlled diabetes mellitus. Although first described in 1965, less than 200 cases have been reported in the literature [1]. This paucity of cases may be a result of the unfamiliarity of this rare condition to clinicians. The patient described in this case report had uncontrolled diabetes mellitus with associated microvascular and macrovascular complications which have been reported previously as risk factors for developing DMI [2]. The average age of presentation for DMI has been reported to be 40 years with a range between 13 and 81 years [3, 4]. Patients with DMI usually present with acute pain with swelling (and occasionally a palpable mass) in an extremity that persists at rest and worsens with exercise, without any prior history of trauma [5]. In addition to the thigh muscles, DMI has also been reported in calf muscles, upper extremity, and abdominal wall muscles [6–8]. Joshi et al. reviewed the clinical course, laboratory findings, and the pathophysiology of DMI in patients on hemodialysis. They noted that in this group of patients DMI was more common in men whereas in most reports DMI is more common in women [7].

Laboratory studies generally demonstrate an elevated ESR and normal or mildly elevated WBC counts. Measurements of creatine kinase (CK, a marker of muscle necrosis) may be normal or elevated depending on timing of blood sampling and amount of muscle involved [7]. In most cases where a normal CK has been reported, there was a delay of several days to weeks after the onset of symptoms before the initial CK level was obtained. A normal CK level thus does not preclude the diagnosis of DMI and is hence not a reliable marker in DMI. Obtaining a CK level within few days after the acute infarct will more likely result in elevated levels. Patient described in this case report had a CKMB level within normal limits. However, his troponin was found to be mildly elevated which is likely secondary to his chronic kidney disease.

The differential diagnosis for focal extremity pain in patients with DM is shown in Table 1. An MRI is often helpful but may not distinguish all of these conditions except amyotrophy, in which case it is essentially normal. Patients presenting with pyomyositis typically have a history of trauma with a staph infection, and an acute presentation. Unlike DMI, they also have an elevated WBC and ESR, normal CK, a purulent aspirate with positive cultures and contiguous areas of involvement on an MRI. Necrotizing fasciitis is a fulminate life-threatening infection, more common in patients with diabetes, that requires immediate surgical intervention if suspected. Patients with tumors usually have a nontender mass with pain developing insidiously.

Since patients with diabetic muscle infarction usually have a stereotyped presentation, it is imperative to recognize this uncommon condition. Venous Doppler and venography are used with the aim of excluding venous thrombosis. MRI is the diagnostic test of choice for diabetic muscle infarction. Due to its superior soft tissue contrast, it is the most sensitive imaging study in detecting DMI. MRI findings demonstrate diffuse edema and swelling of multiple thigh or calf muscles, often in more than one compartment [6].

Muscle biopsy can firmly establish the diagnosis. However, open excisional surgical biopsy is not recommended since it may lead to an increase in healing time due to postoperative complications such as hematoma, seroma, and delayed wound healing [3]. Needle biopsy may be an option in patients with atypical clinical presentation or in whom appropriate treatment measures fail [8]. Muscle biopsy generally demonstrates muscle fiber necrosis, inflammatory cell infiltrate, and microvascular abnormalities.

The underlying pathophysiology of DMI remains incompletely understood although stenosis of intramuscular vessels leading to muscle infarction has been proposed to play a role [9]. Diabetic microangiopathy, in association with hypoxia-reperfusion injury, has also been implicated resulting in severe inflammatory response, edema, and reperfusion [10]. In addition, several other reports have implicated a hypercoagulable state associated with antithrombin II deficiency, increased factor VII, hyperhomocysteinemia, presence of antiphospholipid antibodies, and decreased prostacyclin and tissue plasminogen activator levels as a possible cause of diabetic muscle infarction [11, 12].

The mainstay of treatment for DMI involves early recognition, bed rest, analgesics, and aggressive control of DM. Other modalities suggested to be beneficial include low-dose aspirin, pentoxiphylline, nifedipine, dipyridamole, nonsteroidal agents, and anticoagulants however; there are no randomized control trials to support the use of these agents [13]. Vigorous physical therapy should be avoided since it may lead to an exacerbation [5]. Patients usually recover spontaneously over a period of weeks to months of bedrest although the recurrence rate in the same or the contralateral extremity is approximately 40% in all treatment groups [13, 14]. 

In general the short-term prognosis is reported to be benign, however long-term outcome is discouraging. Patients have been reported to have died within two years after being diagnosed with DMI mostly from the vascular complications associated with diabetes mellitus [14]. In one study the prognosis of DMI in patients with DM is reported as being similar to that after myocardial infarction, with both conditions invariably involving significant vascular injury [15].

## 4. Conclusion

Diabetic muscle infarction is an extremely rare but significant complication of diabetes mellitus with an overall poor long-term outcome. With the increasing prevalence of diabetes mellitus in our society, the incidence of diabetes muscle infarction can be expected to increase. Clinicians need to have a high index of suspicion to recognize this rare condition. With a good clinical history and timely imaging studies, the diagnosis of this rare condition can be made with confidence leading to appropriate intervention and better outcomes.

## Figures and Tables

**Table 1:** Differential diagnosis of focal extremity pain in patients with diabetes mellitus.

(i) Inflammatory: focal myositis, polymyositis
(ii) Vascular: hemorrhage, diabetic muscle infarction, arterial occlusion, thrombophlebitis, lymphedema
(iii) Infectious: pyomyositis, osteomyelitis, cellulitis, necrotizing fasciitis
(iv) Trauma: muscle tear, ruptured cyst
(v) Neoplastic: benign tumors (lipomas, chondromas, and fibromas), sarcomas (liposarcoma, fibrosarcoma)
(vi) Miscellaneous: diabetic amyotrophy, and calciphylaxis
